# Semen Abnormalities, Sperm DNA Damage and Global Hypermethylation in Health Workers Occupationally Exposed to Ionizing Radiation

**DOI:** 10.1371/journal.pone.0069927

**Published:** 2013-07-29

**Authors:** Dayanidhi Kumar, Sujith Raj Salian, Guruprasad Kalthur, Shubhashree Uppangala, Sandhya Kumari, Srinivas Challapalli, Srinidhi Gururajarao Chandraguthi, Hanumanthappa Krishnamurthy, Navya Jain, Pratap Kumar, Satish Kumar Adiga

**Affiliations:** 1 Division of Clinical Embryology, Department of Obstetrics & Gynecology, Kasturba Medical College, Manipal University, Manipal, India; 2 Department of Radiotherapy, Kasturba Medical College, Mangalore, India; 3 Department of Radiotherapy and Oncology, Kasturba Medical College, Manipal, India; 4 National Centre for Biological Sciences, Bangalore, India; 5 Department of Obstetrics & Gynecology, Kasturba Medical College, Manipal University, Manipal, India; Clermont-Ferrand Univ., France

## Abstract

**Background:**

Cytogenetic studies have demonstrated that low levels of chronic radiation exposure can potentially increase the frequency of chromosomal aberrations and aneuploidy in somatic cells. Epidemiological studies have shown that health workers occupationally exposed to ionizing radiation bear an increased risk of hematological malignancies.

**Objectives:**

To find the influence of occupational radiation exposure on semen characteristics, including genetic and epigenetic integrity of spermatozoa in a chronically exposed population.

**Methods:**

This cross sectional study included 134 male volunteers of which 83 were occupationally exposed to ionizing radiation and 51 were non-exposed control subjects. Semen characteristics, sperm DNA fragmentation, aneuploidy and incidence of global hypermethylation in the spermatozoa were determined and compared between the non-exposed and the exposed group.

**Results:**

Direct comparison of the semen characteristics between the non-exposed and the exposed population revealed significant differences in motility characteristics, viability, and morphological abnormalities (*P<*0.05–0.0001). Although, the level of sperm DNA fragmentation was significantly higher in the exposed group as compared to the non-exposed group (*P<*0.05–0.0001), the incidence of sperm aneuploidy was not statistically different between the two groups. However, a significant number of hypermethylated spermatozoa were observed in the exposed group in comparison to non-exposed group (*P<*0.05).

**Conclusions:**

We provide the first evidence on the detrimental effects of occupational radiation exposure on functional, genetic and epigenetic integrity of sperm in health workers. However, further studies are required to confirm the potential detrimental effects of ionizing radiation in these subjects.

## Introduction

The effect of exposure to low levels of diagnostic and therapeutic radiation sources at the workplace is a concern to a large number of health care workers [1]. Ionizing radiation at chronic low doses has been considered as mutagenic and carcinogenic to humans. Cytogenetic studies have demonstrated that even low levels of chronic radiation exposure can potentially increase the frequency of chromosomal aberrations [2],[3] and aneuploidy [4]. Importantly, epidemiological studies have shown that health workers, who are occupationally exposed to ionizing radiation are predisposed to increased risk of hematological malignancies [5],[6].

Reproductive function is sensitive to changes in the physical and chemical environment [7]. However, there is a paucity of data regarding the association between occupational radiation exposure and risk to human fertility [8]–[10]. Evidence from laboratory studies indicate that testicular irradiation in mice can lead to sperm DNA fragmentation [11], which may result in a variety of checkpoint responses in early embryos [12],[13] and transgenerational genomic instability in the offspring [11].

DNA methylation global methylation spermatogenesis have important implications for gamete integrity and transmission of epigenetic information to the offspring [14],[15]. Changes in sperm methylation pattern, induced by toxic exposure, may have widespread repercussions on chromatin integrity and gene expression [16]. Hence, there is a risk of damage to the reproductive system from occupational radiation exposure with adverse implications for fertility and reproductive outcome [17].

In light of these considerations, this study was planned to investigate the possible influence of occupational radiation exposure on semen characteristics including genetic and epigenetic integrity of spermatozoa in a population chronically exposed to radiation.

## Methods

### Study Populations

This cross sectional study conducted between January 2010 and March 2012 comprised of 134 male volunteers, of whom 83 were occupationally exposed to ionizing radiation and 51 were non-exposed control subjects. The occupationally exposed volunteers were selected from various hospitals having diagnostic or therapeutic radiation (X/β/γ rays) facilities. The non-exposed volunteers were employees of the same hospitals but were not exposed to above mentioned radiation sources. The volunteers were of the age group 21 to 50 years who are operating instruments having radiation sources for diagnostic or therapeutic purposes for more than a year. All the subjects were considered as chronically exposed to low dose radiation. Subjects suffering from chronic diseases, endocrine illnesses and history of fever during previous three months were excluded from both the groups. Volunteers who fulfilled the criteria were given a questionnaire to obtain information about the duration of stay at their work place, type of radiation source they were exposed to, their life style, history of illnesses, and problems related to reproduction such as incidences of infertility and miscarriage/s in their partners. The questionnaire also included other confounding factors influencing semen quality and sperm DNA integrity such as, smoking, alcohol, diet, etc., (Table 1). No specific time interval was considered between the time of last irradiation and sample collection. Processing and evaluation of the samples of the two groups were performed in the university infertility research laboratory. The study was approved by the Institutional Ethical Committee, Kasturba Medical College and Hospital, Manipal and a written consent was taken from all the volunteers.

**Table 1:** Characteristics of the subjects included in the study.

Subject characteristics		Non exposed(N:51)	Exposed(N:83)
Subjects handled X-rays		Nil	60
Subjects handled β and γ-rays		Nil	18
Subjects handled X, β and γ-rays		Nil	5
Alcohol consumption	No	14 (27.5%)	27 (32.5%)
	Occasional	37 (72.5%)	56 (67.5%)
	Everyday	0	0
Smoking	No	35 (68.6%)	57 (67.8%)
	Occasional	5 (9.8%)	9 (10.8%)
	<5 times/day	6 (11.8%)	10 (12%)
	>6 times/day	5 (9.8%)	7 (8.4%)
Diet	Strict vegetarian	7 (13.72%)	7 (8.43%)
	Mixed	44 (86.28%)	76 (91.57%)
Marital & fertility status	Married	12 (23.1%)	21 (25.3%)
	Underwent infertility workup	2 (16.66%)	2 (9.52%)
	History of abortions in partners	3 (25.0%)	5 (23.8%)
Congenital malformations in children		0	0

### Exposure Monitoring

The occupational exposure levels of the subjects was routinely monitored by thermoluminescent dosimeter (TLD) device. The cumulative exposure level of each subject was collected from the radiation safety officer of the respective hospital where the subject was enrolled.

### Semen Sample

Semen samples were obtained between 3–5 days of sexual abstinence by masturbation in sterile containers. Semen analysis was performed within one hour of collection under sterile conditions. Upon completion of liquefaction, the sample was mixed well and evaluated for physical and microscopic characteristics according to WHO criteria [18]. Semen analysis of all the samples was performed by an individual who had five years of experience in performing laboratory analysis of the human ejaculate. The analysis of semen and all other assessments were carried out blindly.

### Single Cell Gel Electrophoresis (Alkaline Comet) Assay

Single cell gel electrophoresis (alkaline comet) assay was performed as described earlier [19] with minor modifications. Briefly, the spermatozoa were suspended in PBS and the sperm density was kept constant by appropriate dilution in order to maintain uniformity in distribution of the spermatozoa during electrophoresis. The sperm suspension was mixed with equal volume of 0.75% low melting agarose (Cat No. A 9414, Sigma Chemical Co, USA) and layered on a slide pre-coated with 1% normal agarose (Cat No. 9539, Sigma Chemical Co, USA). A third coat of agarose was layered over the second layer followed by overnight incubation in lysis solution (2.5 M NaCl, 100 mM disodium EDTA, 10 mM Trizma base, pH 10, 1% Triton X-100, 5% DMSO) under alkaline conditions (pH 10) at 4°C. Subsequently, 10 mM DTT was added to the lysis solution and kept for 2 h to ensure sperm head decondensation. Sperm DNA unwinding was carried out for 20 min followed by electrophoretic separation in a buffer (10 N NaOH, 200 mM EDTA, pH>13), 25 V (VcM = 0.74 V/cm, 300 mA) for 30 min followed by neutralization of slides in 0.4 M Tris HCl buffer for 15 min. The slides were dehydrated in chilled absolute alcohol for 30 min and then stained with ethidium bromide (2 µg/mL). The cells were observed under a fluorescent microscope (Imager-A1, Zeiss, Germany) and image was captured using 40× objective. Each slide was coded to avoid observer’s bias and a minimum of 50 images were captured from each samples randomly avoiding the anode end and the edges of the slides. Damaged sperm attain a shape of comet with the tail region consisting of fragmented DNA and the head region intact DNA. The comet evaluation of the captured images was done using Kinetic Imaging software (Komet 5.5, UK). Manual analysis of individual sperm cell was performed based on its comet size and categorized as intact (no tail), minimum damage (small tail, up to 10%) moderate damage (medium tail, between 10–50%) and severely damaged (large tail, >50%). A minimum of 1000 spermatozoa were assessed from each data point.

### Terminal Deoxynucleotidyl Transferase dUTP Nick End Lebelling (TUNEL) Assay

TUNEL assay was performed according to our previously published methodology [20]. A drop of semen was placed on a poly-L-lysine coated cover slip and allowed to dry at room temperature. The cells were fixed in 4% paraformaldehyde solution for 30 min followed by permeabilization using 0.2% Triton X-100 for 30 min. The spermatozoa were incubated in terminal deoxynucleotidyl transferase and the nucleotide mix labeled with FITC (Apoalert DNA fragmentation assay kit, Cat No. 630108; Clontech, Mountain View, CA) for 1 hour at 37°C in a humidified chamber. The cells were washed, counterstained with propidium iodide (10 µg/mL), and mounted on a glass slide. The TUNEL positive cells which exhibited a strong nuclear green fluorescence under a fluorescence microscope (Imager-A1; Zeiss, Gottingen, Germany) were assessed. A total of 2,000 spermatozoa were assessed from each subject and expressed as percentage of TUNEL positive spermatozoa.

### Flow Cytometry Based Sperm Chromatin Integrity Assay

The Sperm Chromatin Structure Assay was performed as described earlier [21]. The sperm density in the ejaculate was adjusted to a concentration of approximately 1–2 million/ml. After removing seminal plasma by repeated washing, the sperm samples were fixed in 70% ethanol and stored until analysis. Prior to analysis, the sperm samples were washed in TNE buffer and treated with a lowpH (pH 1.2) acid-detergent solution containing 0.1% Triton X-100, 0.15 mol/L NaCl and 0.08 mol/L HCl for 30 seconds followed by staining with a final concentration of 6 µg/mL purified Acridine Orange (AO, Cat No 74395, Sigma Chemical Co, USA) prepared in Tris NaCl EDTA (TNE) buffer, pH 6.0 [22]. AO stained sperm were analyzed on a FACS Aria I flow cytometer (Becton Dickinson, San Jose, CA), equipped with a 100 mW air-cooled, solid state 488 nm laser. The green and the red fluorescence signals were collected using band pass filters 525/50 and 585/15 respectively on a linear scale. A minimum of 5000 events were collected per sample. SCSA qualifies the shift from double-stranded to single-stranded DNA following acid denaturation. The extent of denaturation is quantitatively denoted by the term alpha t (αt), a value that can range from 0 to 1. The αt value is a ratio of red fluorescence to total (green and red) fluorescence, and is calculated as follows: mean channel of red fluorescence/mean channel of red fluorescence+mean channel of green fluorescence. A higher shift signifies greater DNA denaturability and reduced/loss of fertility [23].

### Fluorescence *in situ* Hybridization (FISH)

In contrast to other assays used in this study, sperm aneuploidy assessment was performed only on 23 subjects based on the mean cumulative absorbed dose of the individuals (5.43±1.01 mSv, N = 12; <0.05 mSv, N = 11). The *in situ* hybridization was performed as described by Sarrate and Anton [24] with minor modifications. The slides containing spermatozoa were air dried followed by fixation in freshly prepared Carnoy’s fixative (methanol: acetic acid, 3∶1). Sperm decondensation was achieved using 25 mM dithiothreitol dissolved in lysis solution for 5 min at room temperature followed by washing with 2×saline-sodium citrate (SSC) buffer. Air dried slides were immersed in pre-treatment solution (2×SSC, pH 7.4) at 73°C for 2 min and then treated with protease solution (Pepsin, Cat. No. P7012; Sigma Chemical Inc. USA) dissolved in 10 mM HCl for 15 min followed by dehydration using serial graded ethanol solutions. The FISH probes (AneuVysion Multicolor DNA Probe Kit, Vysis CEP 18, X, Y-alpha satellite, LSI 13 and 21, Abbott Molecular Inc. USA) were added onto the cells and denatured at 73°C for 5 min followed by hybridization for 16 h at 37°C in a hybridization chamber (Thermobrite, Abbott molecular, USA). The slides were placed in 2×SSC/0.1% NP-40 at room temperature for 1 min and agitated. The slides were washed in 0.4×SSC/0.3% NP-40 at 73°C for 2 min followed by washing in 2×SSC/0.1% NP-40 at room temperature for 1 min and then counter stained with DAPI. The slides were observed using appropriate filter in/of fluorescent microscope (Imager-A1, Zeiss, Germany) at 100× magnification. All the slides were coded during microscopic evaluation to avoid observer’s bias.

### 5-Methylcytosine Immuno Detection

Immunostaining was performed according to the method described by Tavalaee et al. [25] with minor modifications. The slides containing spermatozoa were air dried followed by fixation in 4% paraformaldehyde (PFA) for 20 min. The cells were washed in PBS and decondensation was done in 25 mM DTT followed by denaturation with 6 N HCl. The spermatozoa were treated with monoclonal anti-5-methylcytosine (5-MEC) antibody (Cat. No. NA81, Calbiochem) at a dilution of 1∶50 and incubated overnight at 4°C in a moist chamber. After washing with PBS, the cells were incubated with FITC labeled Goat anti-mouse IgG (Cat. No. D0408, Santa Cruz), at a dilution of 1∶50, for one hour at 37°C. The cells were counterstained with propidium iodide and observed under fluorescence microscope (Imager-A1, Zeiss, Germany) at 100×magnification. Spermatozoa fluorescing green were considered as hypermethylated. Minimum of 2,000 spermatozoa were scored from each subject.

### Statistical Analysis

The data were analyzed using Statistical Package for Social Sciences (SPSS 15.0). Data has been summarized using mean and standard error (Mean ± SEM) for continuous variables and percentages for qualitative variables like alcohol and smoking. Comparison has been done using independent ‘t’ test for continuous variables and chi-square test for categorical variables. The graphs were plotted using SPSS 15.0.

## Results

### Characteristics of Study Population

The participation rate in the present study was 82.17%. The mean age of the non-exposed and exposed subjects was 28.03±0.83 and 27.74±0.75 years respectively and the difference was not statistically significant between the two groups. The exposed group had an average work experience of 6.51±0.66 years. The history of smoking and alcohol consumption did not differ significantly between the two groups. Further, the incidence of infertility and abnormal reproductive outcome in the spouses of exposed subjects were not significantly different from that of non-exposed subjects spouses (Table 1).

### Semen Characteristics

In total, 134 persons who provided semen sample were included in the study. A direct comparison of the semen characteristics between the exposed and non-exposed populations is shown in Table-2. Although, the ejaculate volume and sperm concentration were not significantly different between the exposed and non-exposed groups, the motility characteristics, especially total (Grade a+b+c) and rapid progressive (Grade c) motility were markedly different between the two groups (*P<*0.001 and 0.01 respectively). Further, the sperm viability was also significantly compromised in the exposed group (*P<*0.05). A significant decline in morphologically normal spermatozoa was observed in the exposed group (*P<*0.0001). The defects are more localized in the sperm head when compared to rest of the structural abnormalities. An analysis of the sperm head vacuoles between the two groups revealed a significantly higher incidence of vacuoles in the exposed group (*P<*0.001).

**Table 2:** Semen characteristics in the health workers exposed to ionizing radiation.

Parameters	Non-exposed(Mean ± SEM)	Exposed(Mean ± SEM)	P Value
Semen volume	2.59±0.19	2.26±0.16	NS
Sperm count (millions/mL)	68.44±5.98	64.16±4.40	NS
Sperm count (millions/ejaculate)	168.72±20.78	152.54±16.39	NS
Sperm motility (%)			
*Grade a+b+c*	65.21±1.80	59.14±1.34	<0.001
*Grade b*	36.04±1.32	37.86±1.46	NS
*Grade c*	19.32±1.77	13.76±1.47	<0.01
Morphology (%)			
*Normal forms*	29.78±1.66	16.94±0.99	<0.0001
*Head abnormality*	66.37±2.07	80.02±1.11	<0.0001
*Amorphous head*	17.96±1.27	21.71±1.32	NS
*Tail defects*	1.95±0.35	2.84±0.37	NS
*Vacuoles*	19.72±2.25	33.35±3.28	<0.001
Viability (%)	65.42±1.77	59.54±2.0	<0.05

### Sperm DNA Integrity

**Figure 1:**
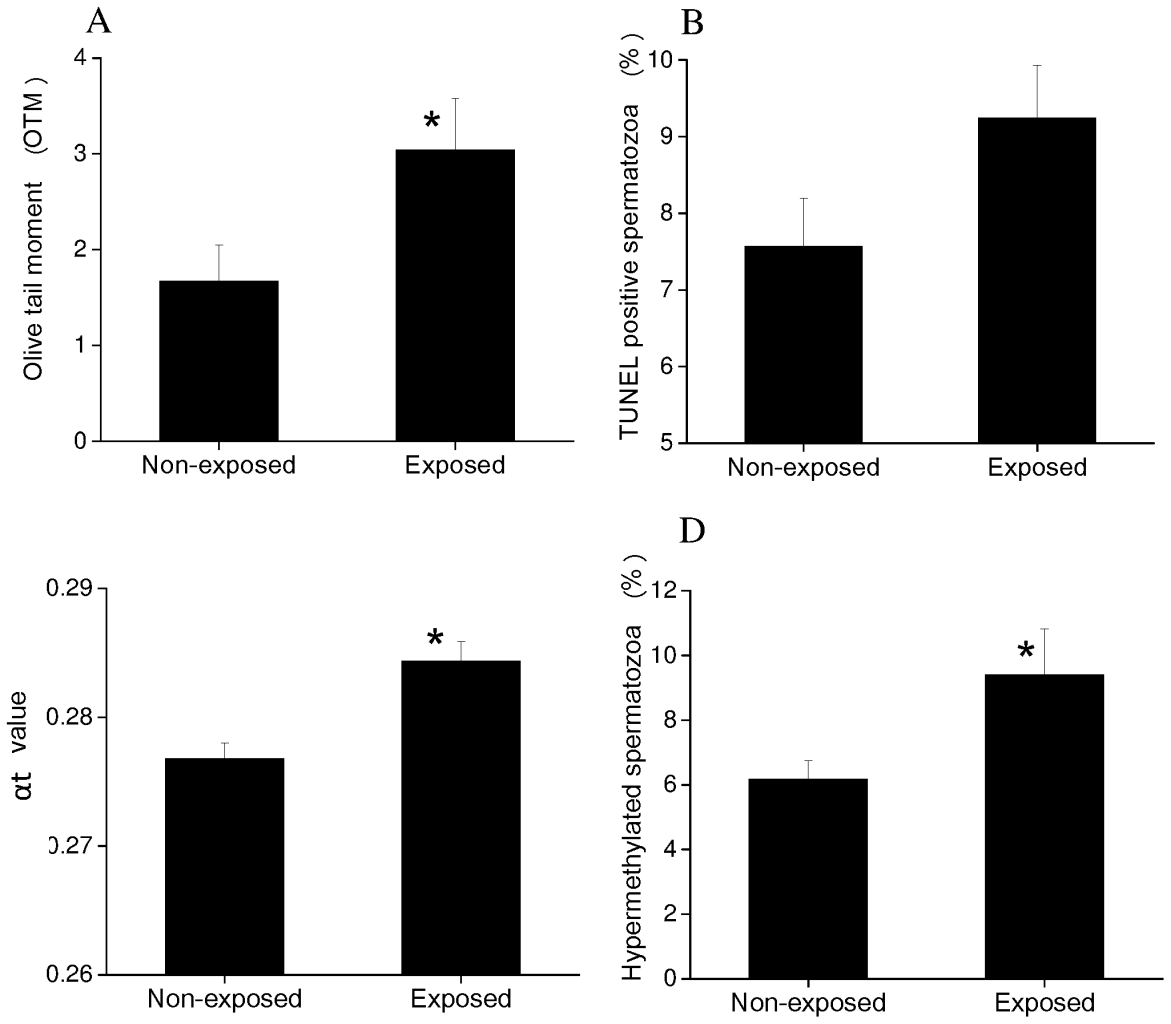
Graphs showing the sperm DNA fragmentation and global methylation status in subjects exposed to ionizing radiation at workplace. (A) Relative OTM level in non-exposed and exposed group. (B) Incidence of percent TUNEL positive spermatozoa. (C) αt values as measured by flow cytometry based sperm chromatin structure assay (D) Percentage of hypermethylated spermatozoa in non-exposed and exposed group.

Alkaline comet assay was performed to quantify the amount of single and double strand DNA breaks in the spermatozoa of occupationally exposed subjects. The olive tail moment (OTM, product of the tail length and the fraction of total DNA in the tail) in the exposed group was approximately 1.8 fold higher than that of the non-exposed subjects (*P<*0.05) (Fig. 1A). Similarly, the percentage of head and tail DNA were also significantly different between the two groups (Table 3). Manual analysis of comet in approximately 1000 spermatozoa demonstrated a significant difference (*P<*0.05–0.0001) in the extent of DNA fragmentation between non-exposed and exposed subjects (Table 3). Although, the TUNEL results are in agreement with comet data, the TUNEL positive cells are not statistically significant between the groups (Fig. 1B, Table 3). Since flow cytometry based sperm chromatin assay provides independent measurement of sperm DNA integrity, it is considered as a useful tool for epidemiological studies [26]. Therefore, it was used for the validation of comet and TUNEL results. A significantly higher αt value was observed in the exposed group when compared to the non-exposed group (*P<*0.0001) (Fig. 1C, Table 3).

**Table 3:** Sperm DNA integrity analysis in the health workers occupationally exposed to ionizing radiation.

Test	Parameters	Non exposed	Exposed	P Value
Comet assay	Olive Tail Moment (OTM)	1.67±0.38	3.05±0.54	<0.05
	Percent Tail DNA	10.44±0.87	15.01±1.24	<0.01
	Percent Head DNA	89.56±0.87	84.99±1.24	<0.01
Comet assay (manual analysis)	Intact DNA (%)	68.42±4.32	49.61±2.95	<0.001
	Minimum DNA damage (%)	14.56±1.75	24.82±1.83	<0.0001
	Moderate DNA damage (%)	6.23±1.73	10.77±1.18	<0.05
	Severe DNA damage (%)	10.79±3.49	14.8±3.17	NS
TUNEL	TUNEL positive sperm (%)	7.57±0.63	9.24±0.69	NS
Sperm Chromatin Structure Assay	αt value	0.2766±0.0012	0.2844±0.1115	<0.0001

### Incidence of Sperm Aneuploidy

Sperm aneuploidy assessment was performed on 23 exposed subjects, of which 12 subjects had the mean cumulative absorbed dose of 5.43±1.01 mSv. The mean absorbed dose of the remaining 11 subjects was <0.05 mSv.Therefore, this subgroup was considered as the internal control. Except the exposure level, other confounding factors such as smoking and alcohol consumption were not significantly different between the two groups. A minimum of 5000 spermatozoa from each subject were evaluated for each chromosome to determine the incidence of disomies/trisomies. The iincidences of disomy in chromosomes 13, 18, 21, X and Y was not significantly different between the groups studied, although, the overall incidence of aneuploidy was moderately high in the exposed group (Table 4).

**Table 4:** Incidence of sperm aneuploidy in the health workers exposed to ionizing radiation at workplace.

Chromosome	Group	Sperm (N)	Normal	Disomies	Trisomies	Incidence (%)	P Value
13	<0.05 mSv	9541	9513	28	0	0.29	
	≥0.50 mSv	10411	10385	26	0	0.24	0.64
18	<0.05 mSv	10324	10296	27	1	0.27	
	≥0.50 mSv	11202	11165	37	0	0.33	0.42
21	<0.05 mSv	9468	9445	23	0	0.24	
	≥0.50 mSv	10651	10612	39	0	0.36	0.14
X	<0.05 mSv	11000	10976	24	0	0.21	
	≥0.50 mSv	12000	11972	28	0	0.23	0.91
Y	<0.05 mSv	11000	10978	22	0	0.20	
	≥0.50 mSv	12000	10963	37	0	0.30	0.13
XY	<0.05 mSv	5201	5199	2	0	0.03	
	≥0.50 mSv	5305	5302	3	0	0.05	0.68
All	<0.05 mSv	56534	56407	126	1	0.22	
	≥0.50 mSv	61569	61399	170	0	0.28	0.07

### Global Methylation in Spermatozoa

The global methylation analysis as measured by the number of hypermethylated spermatozoa were significantly higher in the exposed group when compared to the non-exposed population (*P<*0.05) (Fig. 1D).

### Influence of Confounding Factors

To rule out the effect of confounding factors on the present outcome, we performed a cross tabulation of the smokers and alcoholics in both the exposed and non-exposed groups. The percentage of smokers and alcoholics was found to be similar in both the groups (Table-1). The chi-square value of smokers and alcoholic groups were 0.995 and 0.535 respectively which was not significant. The analysis of covariance (ANCOVA) using SPSS has ruled out the influence of these potential confounding factors on the results observed in the present study.

## Discussion

While earlier studies have clearly demonstrated the detrimental effects of occupational radiation exposure on the integrity of somatic cells, its impact on semen characteristics, including genetic and epigenetic integrity of male gametes, has not yet been elucidated. The present study, for the first time, indicates that occupational radiation exposure may be associated with alterations in human semen quality. The most pronounced effects observed in spermatozoa of exposed subjects were changes in motility characteristics, increased sperm morphological abnormalities, sperm DNA fragmentation and global hypermethylation. These findings show that exposure to occupational radiation may have a profound implication on the fertility and reproductive outcome of health workers, and importantly, on the health of the children born to such fathers since the spermatozoa carrying nuclear abnormalities can fertilize the oocytes [27], and the embryos thus derived from the irradiated sperm carry substantial risk of transgenerational genomic instability [11], [12].

It is now evident that well controlled semen evaluation studies have contributed substantially to current knowledge on reproductive toxicity of many chemicals in humans [28]. Sperm concentration, which is the most important determinant of male fertility [29], did not vary significantly between the exposed and non-exposed groups eventhough the ejaculatory abstinence of 3–5 days was maintained for all the subjects. The evaluation of sperm motility is useful when a toxicant is expected to influence the percentage of motile spermatozoa or sperm motility pattern. Prolonged exposure to high levels of testicular toxicant may produce testicular atrophy thereby shutting off spermatogenesis. However, at lower levels, the adverse effect may be limited to only changes in the motility pattern without affecting fertility significantly [30]. The International Commission on Radiological Protection (ICRP), in its 2007 recommendations based on occupational classification, limits artificial irradiation of the public to an average of <1 mSv of effective dose per year, and ≤6 or ≤20 mSv/year in the case of occupationally exposed subjects [31]. Since occupational radiation exposure levels now strictly fall well within the accepted limits [32], the long term consequence of low level exposure is possibly observed as altered motility characteristics without affecting the sperm output in these subjects. Hence, from a clinical point of view, it is possible that the risk of occupation induced infertility arising from deteriorated semen quality is substantially lower in exposed subjects since the semen characteristics of exposed men in the present study were above the WHO recommended threshold level [18], [33].

It has been revealed that, sperm morphology is the best predictor of fertilization potential [34]. A high proportion of morphologically abnormal spermatozoa in the exposed group, therefore indicates the possible association between chronic radiation exposure and defective spermiogenesis. Our observations are in agreement with the results from an earlier study which demonstrated increased sperm ultra-structural defects in Chernobyl salvage workers exposed to radiation [17]. Apart from morphological abnormalities, the exposed subjects in the present study also had an increased incidence of sperm head vacuoles. It has been shown that large vacuoles in spermatozoa are linked to failure in chromatin condensation and subsequent nuclear weakness [35], which in turn leads to sperm DNA damage [36].

The evidence of a relationship between sperm morphological abnormality and their abnormal cytogenetic content [37] prompted us to examine the sperm chromosomal integrity of the exposed subjects. Despite increased incidences of structural and numerical chromosomal abnormalities in the somatic cells of health workers exposed to radiation [4], [38], the present data did not demonstrate significant differences in sperm aneuploidy level between the two groups.

Earlier studies have indicated that environmental toxicants can potentially induce sperm DNA fragmentation [39]. Results of experimental studies have shown that sperm carrying DNA damage is capable of fertilizing an oocyte [26],[40] however, viability is compromised in the embryos derived from the irradiated sperm [40].

Importantly, embryos derived from irradiated sperm show unique DNA damage response pathways during their preimplantation development [12], [13]. Radiation induced aberrant DNA repair process carry the risk of transgenerational genomic instability in both somatic and germ cell compartments in the offspring [11]. Hence, the present study laid emphasis on an independent critical assessment of sperm DNA integrity in the exposed group using three well established techniques. It has been shown that alkaline comet assay is an additional complement to standard biodosimetric methods for the detection of cytogenetic risk in radiation exposed health workers [41], [42]. The comet analysis in the present study has revealed an increase in DNA fragmentation in the spermatozoa of exposed subjects. These results are in agreement with an earlier study which reported a similar comet pattern in peripheral lymphocytes [42]. In addition, our TUNEL data also suggests an increased level of sperm DNA fragmentation although statistical power could not be established. In contrast, the DFI as measured by flow cytometry has demonstrated a strong statistical power between the non-exposed and the exposed group. However, correlation between individual assays was not attempted since the damage detection techniques applied here measure different features, and hence vary in their specificity. Nevertheless, from the clinical point of view, it is important to know that sperm carrying fragmented DNA still have the ability to fertilize an oocyte [26] and that sperm DNA integrity is associated with male fertility potential **in vivo** and **in vitro**. Notably, natural fertility potential is compromised in ejaculates containing a high percentage of DNA damaged spermatozoa [22], [43]. Apart from an abnormal post-implantation embryonic development, sperm DNA fragmentation may compromise progression of pregnancy, resulting in spontaneous miscarriage following assisted conception [44]. Therefore, consequences of increased DNA fragmentation in exposed subjects’ needs to be addressed in relation to their reproductive competence.

Radiation induced aberrant DNA methylation may play a role in the predisposition to pathological states and disease development [45]. It has been shown that radiation induced epigenetic changes may arise in the cell without initiating chromosomal instability [46]. Nonetheless, increased morphological abnormalities, vacuoles and DNA fragmentation in the spermatozoa of exposed subjects may be linked to the sperm epigenetic changes due to destabilization of nucleosomes prior to histone-protamine conversion and an eventual rise in DNA methylation levels [47]. Thus, increased global hypermethylation observed in the exposed group implies defective chromatin condensation resulting in morphologically abnormal spermatozoa. It is possible that alteration in methylation is transmitted to subsequent generations providing a persistent epigenetic signal [16] which raises the concern on the hypermethylation status, and the eventual adverse effect on reproductive outcome in these subjects. Importantly, parental exposure to radiation may induce epigenetic alterations which eventually play a pivotal role in the molecular etiology of transgenerational genome instability [48]. Although, global DNA methylation analysis may not be capable of detecting subtle epigenetic variations in individual gene regions, it can give a general overview on methylation status. This highlights the need for further analysis of specific imprinted genes to elucidate the repercussions of methylation changes observed in this study.

Well-designed epidemiological studies can provide information about the risks to sperm structure, genetic and epigenetic integrity, posed by environmental contaminations [38]. The present study is unique in its examination of the possible influences of radiation exposure. The strengths of the present study are: a) the use of highest number of exposed subjects than any of the earlier reports on radiation exposed health workers, and b) simultaneous evaluation of functional, genetic and epigenetic integrity of male gametes. In addition, only one technically competent individual was involved in analyzing the semen characteristics to reduce the potential impact by inter-observer variation. Further, selection bias is unlikely since the age of the subjects did not differ between two groups and the non-exposed subjects were recruited from other departments of the same hospitals. It has been shown that confounding factors such as smoking and alcohol can influence genetic damage induced in humans by ionizing radiation [31] possibly by increasing the radio sensitivity of the cells [49]. However, cross-tabulation analysis of smokers and alcoholics did not reveal any significant difference between the two groups which excludes the possible influence of confounding factors on our results.

In conclusion, the present data clearly suggests that occupational radiation exposure may have substantial detrimental effect on sperm functional, genetic and epigenetic integrity in health workers. Due to limited sample size, we did not find any significant differences in terms of infertility and abnormal reproductive outcomes in the spouses of exposed subjects, however, future studies are certainly needed in large population to address the reproductive fitness of the exposed individuals and also the health status of the children born to radiation exposed health workers.
